# Functional traits partially mediate the effects of chronic anthropogenic disturbance on the growth of a tropical tree

**DOI:** 10.1093/aobpla/ply036

**Published:** 2018-06-05

**Authors:** Isidore O Amahowe, Orou G Gaoue, Armand K Natta, Camille Piponiot, Irié C Zobi, Bruno Hérault

**Affiliations:** 1 Faculty of Agronomy, University of Parakou, Parakou, Benin; 2 Department of Ecology and Evolutionary Biology, University of Tennessee, Knoxville, TN, USA; 3 Department of Geography, Environmental Management and Energy Studies, University of Johannesburg, APK Campus, Johannesburg, South Africa; 4 Cirad, UMR EcoFoG (AgroParistech, CNRS, Inra, Université des Antilles, Université de Guyane), Campus Agronomique, Kourou, French Guiana; 5 Institut National Polytechnique Félix Houphouët-Boigny, Yamoussoukro, Ivory Coast

**Keywords:** Debarking, growth performance, pruning, resilience strategy, wood density

## Abstract

Understanding how trees mediate the effects of chronic anthropogenic disturbance is fundamental to developing forest sustainable management strategies. The role that intraspecific functional diversity plays in such process is poorly understood. Several tree species are repeatedly defoliated at large scale by cattle breeders in Africa to feed livestock. In addition, these tree species are also debarked for medicinal purposes. These human-induced disturbances lead to biomass loss and subsequent decline in the tree growth. The main objective of this work is to investigate how functional traits mediate tree response to chronic anthropogenic disturbance. We used a unique data set of functional traits and growth rate of 503 individual tree of *Afzelia africana*. We collected data on leaf mass per area (LMA), wood density (WD) and growth rate, and recorded history of human disturbances (debarking, pruning) on individual tree from 12 populations of *A. africana* distributed in two ecological zones in Benin (West Africa). We tested the effect of disturbances on absolute growth rate across ontogenetic stages, assessed the role of intraspecific trait variability on growth and tested the role of tree functional strategy on the tree growth response to debarking and pruning. We found that debarking did not affect stem growth, suggesting a fast compensatory regrowth of bark wounded. Moreover, tree response to debarking was independent of the functional strategy. By contrast, we found that pruning reduced tree absolute growth; however, trees with low WD were more strongly affected by pruning than trees with high WD. Our results emphasize the importance for plant functioning of the interplay between the availability of leaves for resource acquisition and a resilience strategy by mobilizing stored resources in stem wood to be reinvested for growth under severe disturbances.

## Teaser

This study demonstrates that functional traits can mediate the response of a tropical tree to chronic anthropogenic disturbance. Pruning by indigenous Fulani people, a cultural practice in West African savanna, can reduce tree growth. From a conservation perspective, tree location also affected their functional traits indicating that even classical conservation measures can affect tree ecology. Trees that are in protected areas where indigenous Fulani people do not have access tend to have heavier wood density (WD). We showed that these trees with higher WD are most resilient to branch pruning.

## Introduction

Functional traits can aid in our understanding of the variation in species response to environment (McGill *et al.* 2006). Wood density (WD) and leaf mass per area (LMA) are key plant traits that define resource allocation strategies for essential functions (acquisition, storage), and particularly reflecting the trade-offs between construction cost of new plant structures (leaf or stem wood) and survival under different conditions of the environment (Poorter *et al.* 2008, 2010; Iida *et al.* 2014). First, Poorter *et al.* (2008) examined the relationship between functional and demographic rate for 240 tree species in Neotropical forests, and found that growth and mortality rate decreased with increasing WD, suggesting a trade-off between stem construction cost and resistance to damage. Second, with high LMA, leaves exhibit high thickness and toughness that reflect greater resource allocation into defence than into growth (Wright and Cannon 2001). This leads to more robust leaves with long lifespan (Sterck *et al.* 2006) and therefore less damaged from herbivory (Wright and Westoby 2002).

Most studies linking functional traits to growth rates have focused on the interspecific trait variability as a convenient proxy of the ecological strategies which vary largely among coexisting species (Westoby *et al.* 2002). However, our understanding of intraspecific functional trait variation and how this mediates plant demography response to environmental variation and disturbance is limited (Poorter *et al.* 2008; Martínez-Vilalta *et al.* 2010; Wright *et al.* 2010; Albert *et al.* 2011; Iida *et al.* 2014). Furthermore, several studies independently demonstrated ontogenetic variation in plant growth/survival and functional traits (Poorter and Bongers 2006; Hérault *et al.* 2011; Iida *et al.* 2014). For example, growth, survival and reproduction vary positively with individual size or age (Ellner and Rees 2006; Zuidema *et al.* 2010; Gaoue 2016). In addition, functional traits such as WD, seed mass and adult stature are good predictors of plant growth and/or mortality (Poorter *et al.* 2008). However, the role of ontogeny in the relationship between functional traits and demography is rarely examined with most studies focusing on a single life stage, such as sapling or adult (Poorter *et al.* 2008). Recent studies have highlighted the importance of considering ontogenetic shifts in the relationship between functional traits and disturbance (e.g. Herault *et al.* 2010).

In tropical regions, many tree species are debarked or pruned for subsistence, medicinal and economic purposes (Ouédraogo-Koné *et al.* 2006; Gaoue and Ticktin 2007; Nacoulma *et al.* 2017). These types of damage alter tree ecophysiological functioning (Gaoue *et al.* 2011) and can ultimately reduce the demographic performance of harvested plants (Rijkers *et al.* 2006; Gaoue *et al.* 2011, 2013). Stem bark wound is a direct biomass removal of an important plant tissue that induces shift in internal resource allocation patterns and often leads to a decrease in tree growth (Bazzaz *et al.* 1987), even if compensatory growth may sometimes occur when bark removal is not recurrent (Gaoue *et al.* 2011). However, the wounded organ can recover through several internal mechanisms (Ryan and Yoder 1997) depending on the debarking intensity and the intrinsic recover ability of the species (Gaoue and Ticktin 2007; Delvaux *et al.* 2010).

Pruning trees creates significant branch and leaf biomass loss, with long-lasting impacts on the whole-plant photosynthesis performance (Anten and Ackerly 2001; Anten *et al.* 2003; Gaoue *et al.* 2011). Consequently, pruning may result into a shortage of the amount of resources allocated for growth. From an evolutionary perspective, many tree species have evolved to cope with repeated disturbances (Gutschick and BassiriRad 2003) and exhibit various recovery processes that are ultimately shaped by the tree functional strategy. Previous studies have shown that the effects of both debarking and pruning are size-dependent (Sinsin *et al.* 2004; Gaoue and Ticktin 2007; Mensah *et al.* 2014; Amahowe *et al.* 2017), because resource availability and quality are linked to the tree life stage. Failure to account for this size-dependent growth, both in tree performance and in assessing the effect of pruning and debarking, may result into suboptimal management decisions.

In this study, we used a unique data set of 503 individual trees from 12 populations of *Afzelia africana*, a tropical tree distributed from the Sudano-Guinean to the Sudanian zones in Benin, to test the effects of pruning and debarking on the absolute growth rate while accounting for the ontogenetic tree growth trajectory. We assessed the effects of intraspecific variability in the leaf and wood economic spectrum on individual tree growth performance and tested the effects of the individual tree functional strategy on tree growth response to debarking and pruning.

## Methods

### Study area

This study was conducted in the Republic of Benin (6°–12°50′ N and 1°–3°40′ E), West Africa. The country covers 114763 km^2^ and comprises three main ecological zones including Guineo-Congolean (6°25–7°30 N), Sudano-Guinean (7°30–9°30 N) and Sudanian (9°30–12° N) ecological zones. The study was conducted in the latter two zones, where *Afzelia* populations were mostly distributed. The physical characteristics of the two zones were reported in Gaoue and Ticktin (2008). Rainfall ranges from 800 to 1300 mm, while temperature varies from 24 to 31 °C. The vegetation is dominated by savannas, woodlands, dry dense forests and gallery forests.

### Study species

*Afzelia africana* is a multi-use tree species belonging to Fabaceae family (Angiosperm Phylogeny Group: APG III). Its crown can spread up to 35 m in height, with a diameter at breast height (DBH) measuring up to 1 m (Arbonnier 2000; Akoègninou *et al.* 2006; Assogbadjo *et al.* 2010). Flowering occurs at the end of dry season (April–May), fruiting in the middle of rainy season and fruits mature in December/January onward (Sacande 2007; Orwa *et al.* 2009). Seeds are dispersed by wind and animals (Orwa *et al.* 2009). In Sudanian and Sudano-Guinean zones of Benin, *A. africana* is distributed in savannas, woodlands, dry dense forests, gallery forests (Mensah *et al.* 2014), edge of dry forest and mountainous zones (Akoègninou *et al.* 2006), while it is found in dense semi-deciduous forest (Bonou *et al.* 2009) in Southern Benin. The species is defoliated by indigenous Fulani people to feed cattle (Ouédraogo-Koné *et al.* 2006; Nacoulma *et al.* 2017), and debarked for medicinal purposes (Kone *et al.* 2004; Delvaux *et al.* 2009; Tshisikhawe *et al.* 2012). In addition to debarking, the species trunk bark is frequently wounded and shows large deep scars. Pruning and debarking intensity are size-dependent (Amahowe *et al.* 2017). The species is sensitive to fire at early age and this is worsened by insect attack (Stark 1986; Delvaux *et al.* 2009).

### Sampling strategy

**Figure 1.**
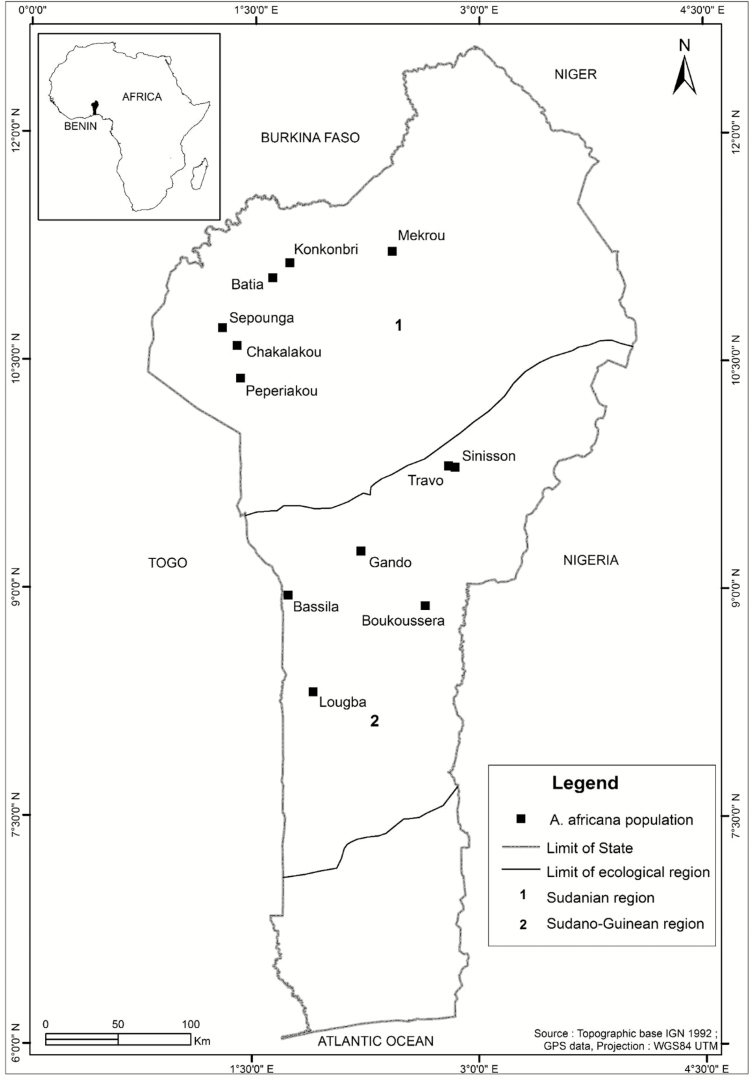
Study area and location of the 12 sampled populations of *Afzelia africana* studied. Number 1 indicates the drier Sudanian zone and ‘2’ indicates the wetter Sudano-Guinean ecological zone.

We randomly sampled 12 populations of *A. africana* out of 52 populations surveyed in Benin during an exploratory study in December 2014. These populations were equally distributed across two main ecological zones: Sudano-Guinean and Sudanian zones. In each zone populations were separated by a minimum of 10 km from each other (Fig. 1). Within each population, 1 ha plot was used to record the DBH for all *A. africana* individuals with DBH ≥ 2 cm. This represents a total number of 503 trees sampled and measured in Sudano-Guinean and in Sudanian zones. Debarking and pruning intensity were assessed on each sampled tree. For each tree, we recorded if it was pruned or not. Pruning intensity was estimated at the tree level as the proportion of branches pruned related to the total number of branches. We also calculated the percentage of area covered by the wound on the trunk up to the first main ramification to assess the debarking intensity as in previous studies (Cunningham 2001; Gaoue and Ticktin 2007).

### Functional trait measurements

To measure WD, we collected wood samples from all the 503 individual of *A. africana* trees using the increment borer that offered cylindrical-shaped wood cores (Chave 2005), after the growth period (December 2014 to December 2015), at the end of rainy season. Then, we measured the length (*L*) and the cross-section diameter (*D*) with a calliper, and subsequently calculated the volume (*V*). Cores were oven-dried up to 100 °C for 5 days then weighed using an electronic balance. Wood density was estimated as a ratio of the wood fresh volume by wood dry mass (Cornelissen *et al.* 2003; Chave 2005; Matilo *et al.* 2013). Wood density is a key trait defining the wood economic spectrum (Chave 2005) often seen as orthogonal to the leaf economic spectrum (Baraloto *et al.* 2010).

Leaf mass per area is a key plant trait of the leaf economic spectrum related to biomass investment (Cornelissen *et al.* 2003) and is a good predictor of growth (Grime *et al.* 1997; Wright *et al.* 2004). To measure LMA, we collected for each tree, three visibly intact and sunlight-exposed leaves discarding any leaf that suffered herbivory (Cornelissen *et al.* 2003). These leaves were scanned on flatbed scanner and each image was processed to calculate leaf area using ImageJ (https://imagej.nih.gov/ij/). Leaves were oven-dried at 60 °C for 72 h, before measuring their dry weight and calculate the LMA.

### Modelling framework

We modelled the ontogenetic absolute growth rate of *A. africana* following a model developed by Hérault *et al.* (2011) to fit a sigmoid curve to tree growth trajectory (Model 0). We then tested for the effects of disturbance (individual tree pruning and debarking, Model 1), intraspecific functional trait variation (Model 2) on tree absolute growth rate and finally the effect of functional strategy on tree absolute growth response to disturbance (Model 3). The model without any effect of disturbance or functional strategy (Model 0) is as follows:

**Model 0**

$$log(AGR_{i}+1)\sim Norm\left(Gmax\;\times \;e^{-\frac{1}{2}{\left(log\left(\frac{DBH_{i}}{Dopt}\right)\right)}^{2}};\;\sigma^{2}\right)$$ (0)

where $\;DBH_{i}$ represents diameter at breast height of individual tree *i* and $AGR_{i}$ is its absolute growth rate, i.e. the difference in $\;DBH_{i}$ for each individual tree *i* measured between two consecutive years, 2014 and 2015. Parameters *Gmax* and *Dopt*, respectively, refer to the maximum value of $log(AGR_{i}+1)$ and the *DBH* value at which this maximum value is reached, and σ^2^ is the variance. The effects of covariates (debarking, pruning and functional traits) were tested on *Gmax*. We did not test for the effects of covariates on *Dopt* and σ because of (i) convergence problems when introducing similar covariates in different part of a model and (ii) no clear biologically relevant hypothesis to test for.

**Model 1**—This model tests the effect of disturbance (debarking, pruning) on tree absolute growth rate includes Model 0 (see equation 0 above) with

$$\;Gmax_{i}=Gmax_{1}+\theta_{D}Debark_{i}+\theta_{P}Prun_{i},$$ (1)

where $Gmax_{1}$ is the value of *Gmax* where there is no disturbance (*Debark* and *Prun* equal to 0), *Debark* and *Prun* are values of, respectively, the debarking and pruning variables (from 0 to 100 %) and $\theta_{D}$ and $\theta_{P}$ the model parameters.

**Model 2**—This model tests for the role of intraspecific trait variability on tree absolute growth rate includes Model 0 (see equation 0) with

$$\;Gmax_{i}=Gmax_{2}+\theta_{WD}WD_{i}+\theta_{LMA}LMA_{i},$$ (2)

where $Gmax_{2}$ is the value of *Gmax* for an average (i.e. mean values of *WD* and *LMA*) tree, *WD* and *LMA* are values of, respectively, the WD and LMA variables and $\theta_{WD}$ and $\theta_{LMA}$ the model parameters.

**Model 3**—This model tests for the importance of the individual functional strategy on tree absolute growth response to disturbance. To avoid over-parameterization, all interaction terms between disturbance (*Debark* and *Prun*) and traits (*WD* and *LMA*) were included sequentially in the model using a forward variable selection procedure. The final model includes Model 0 with

$$\;Gmax_{i}=Gmax_{3}+\theta_{P}Prun_{i}+\;\theta_{WD}WD_{i}+\theta_{P-WD}Prun_{i}WD_{i},$$ (3)

where $Gmax_{3}$ is the value of *Gmax* for an average (i.e. mean values of *WD* and *LMA*) tree with no disturbance (*Debark* and *Prun* equal to 0) and $\theta_{P-WD}$ the model parameter that was retained by the selection procedure and reflected the interaction effect between disturbance Prun and the individual functional strategy (*WD*). All models were written and inferred in a Bayesian framework using Monte Carlo Markov Chain (MCMC) algorithms under the R environment (R Development Core Team 2016).

## Results

**Figure 2.**
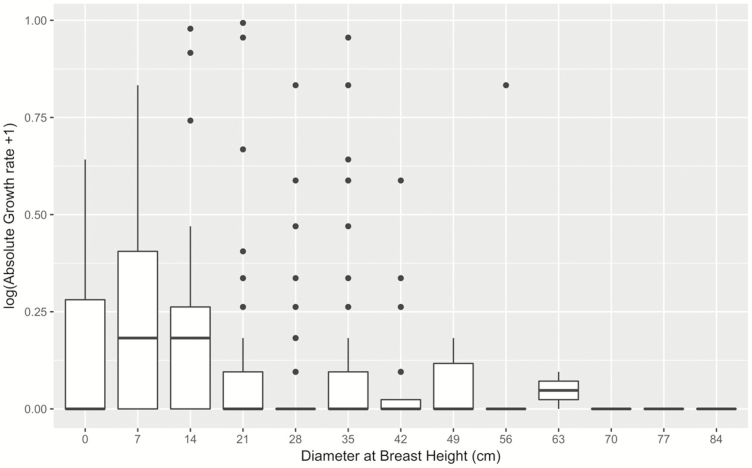
Size-dependent growth rate of *Afzelia africana*. The highest growth rates are obtained at intermediate DBH justifying the use of a hump-shaped growth trajectory to model the effect of stress on tree performance. White rectangles span from the first to the third quartile. A segment inside the rectangle shows the median and black lines above and below the box show the locations of the minimum and maximum. Black dots refer to outliers.

**Figure 3.**
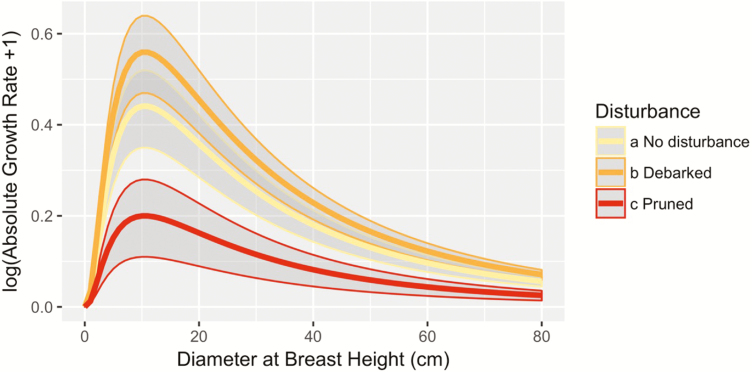
Effect of stress disturbance (debarking and pruning) on the absolute growth rate of *Afzelia africana* (Model 1). Model predictions (lines) are represented for not-pruned (yellow), pruned (red) and debarked (orange) growth trajectories with envelops showing the prediction credibility intervals.

The growth curve of *A. africana* showed a clear hump-shape (Fig. 2). The overall size-dependent model (Model 0) yielded a maximum growth rate of 0.36 cm per year with a diameter at maximum growth of 8.76 cm (Fig. 3, Table 1). We found no significant effect of debarking (Table 1) on maximum growth rate relative to the Model 0 without disturbance (Fig. 3). However, pruning exerted significantly negative effects on absolute growth rate (Table 1, Fig. 3). Pruned trees at optimal DBH, grew at an average of 0.24 cm per year less than non-pruned trees (Table 1, $\theta_{P}=-0.\text{24}$

**Table 1.** Posterior values for the three growth models parameterized in a Bayesian framework. Model 0: the model without any effect of disturbance or functional strategy; Model 1: testing the effect of disturbance (debarking, pruning) on growth performance; Model 2: role for intraspecific trait variability on individual performance; Model 3: importance of the individual functional strategy on the growth response to disturbance. *Gmax* and *Dopt* are, respectively, to the maximum value of $log\text{(}AGR_{i}\text{+1)}$ and the *DBH* value at which this maximum value is reached. $Gmax_{\text{1}}$ is the value of *Gmax* where there is no disturbance (*Debark* and *Prun* equal to 0), and $\theta_{D}$ and $\theta_{P}$ the model parameters. $Gmax_{2}$ is the value of *Gmax* for an average (i.e. mean values of *WD* and *LMA*) tree, and $\theta_{WD}$ and $\theta_{LMA}$ the model parameters. *$Gmax_{3}$* is the value of *Gmax* for an average (i.e. mean values of *WD* and *LMA*) tree with no disturbance (*Debark* and *Prun* equal to 0) and $\theta_{P-WD}$ the model parameter.

	Parameter	Value at maximum likelihood	95 % credibility intervals
Model 0	*Gmax*	0.36	[0.29; 0.43]
	*Dopt*	8.76	[6.77; 11.54]
	*σ*	0.51	[0.48; 0.55]
Model 1	$Gmax_{1}$	0.44	[0.35; 0.52]
	$\theta_{D}$	0.12	[−0.44; 0.69]
	$\theta_{P}$	−0.24	[−0.44; −0.03]
Model 2	$Gmax_{2}$	0.34	[0.24; 0.48]
	$\theta_{WD}$	−0.07	[−0.16; 0.06]
	$\theta_{LMA}$	−0.02	[−0.13; 0.09]
Model 3	$Gmax_{3}$	0.41	[0.27; 0.57]
	$\theta_{P}$	−0.18	[−0.37; 0.02]
	$\theta_{WD}$	−0.10	[−0.22; 0.03]
	$\theta_{P-WD}$	0.12	[−0.09; 0.32]

**Figure 4.**
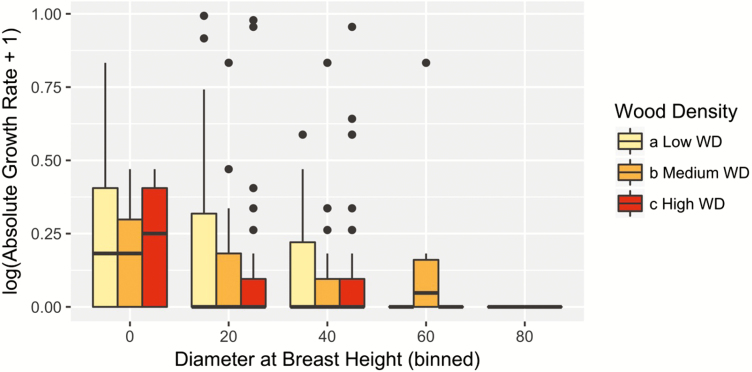
Weak role of intraspecific WD variability in observed growth of *Afzelia africana* (Model 2). Data are binned into low (WD values < quantile 0.33), high (WD values > quantile 0.66) and medium (in between) categories.

**Figure 5.**
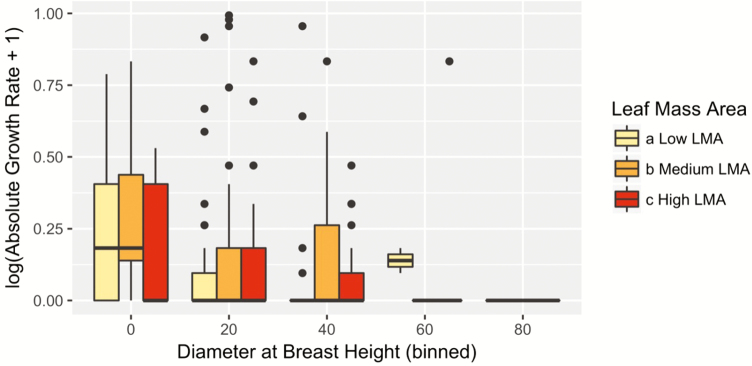
Weak role of intraspecific LMA variability in observed growth of *Afzelia africana* (Model 2). Data are binned into low (LMA values < quantile 0.33), high (LMA values > quantile 0.66) and medium (in between) categories.

**Figure 6.**
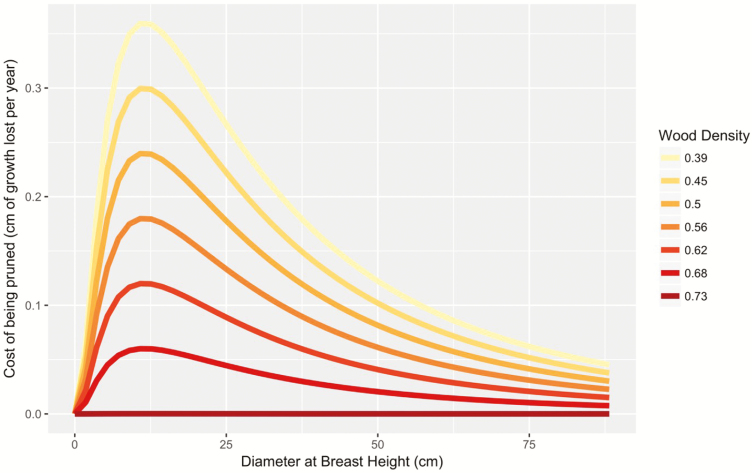
Model predictions showing how the individual WD value mediates the tree response to pruning (Model 3). The cost of being pruned is calculated using the difference in predicted growth between a pruned and a not-pruned tree taking into account both WD and the ontogenetic stage.

Across populations, the mean LMA was 58.17 ± 7.62 g m^−2^ and mean WD was 0.56 ± 0.005 g cm^−3^. When we included the effect of intraspecific functional traits variability in our model (Model 2), we found no significant effect of LMA and WD on growth rate (Table 1, Figs. 4 and 5). However, there was a significant interactive effect $\theta_{P-WD}Prun_{i}WD_{i}$ of WD and pruning on absolute growth rate (Table 1). Pruning significantly reduced the growth of *A. africana* trees by up to 0.35 cm per year, and this negative effect was highest for intermediate aged trees, i.e. around 10 cm with lower WD (Fig. 6). The growth reduction caused by pruning was greater for trees with lower WD than the opposite (Fig. 6). Notably, trees with very dense wood (above 0.70 g cm^−3^), independent of their size, experienced less effect of pruning (close to 0) than trees with low WD. However, this advantage of high WD trees became negligible for very large trees (Fig. 6) which may be due to the low growth rates of these old trees (Fig. 2).

Based on our results, we further tested the hypothesis that trees sampled in populations with low pruning pressure and trees sampled outside forest reserves, where pruning intensity is high, exhibit different population level WD values. We demonstrated that on average, WD was 0.06 g cm^−3^ lower in trees found within forest reserves (Student’s *t*-test, *P* < 0.001).

## Discussion

This study investigated the effect of intraspecific functional trait variation on the growth rate of a tropical tree and how it responds to chronic anthropogenic disturbance. We found a hump-shaped diameter growth curve which reaches a maximum growth of 0.4–0.6 cm per year (Table 1). This maximum growth rate was reached for trees with DBH of 10 cm. Growth rates for trees larger than this threshold were slow. Not surprisingly, trees tend to first grow proportionally to their size given that the largest individuals have more access to light, enhancing photosynthesis (Colbert *et al.* 2002; Motallebi and Kangur 2016). However, when individual trees reach the maturity, they invest more in reproduction, maintenance and defence rather than growth (Coley *et al.* 1985; Ryan and Yoder 1997; Poorter *et al.* 2008). For *A. africana*, the maximum growth rate was reached at smaller diameter values than reported for other tropical trees (Bragg 2002; Karyati *et al.* 2017). For example, in the Amazonian forest, while many canopy species have diameter at maximum growth of 30–50 cm, understory species show lower values (Hérault *et al.* 2011). In South-East Asia, widespread species such as *Endospermum diadenum*, *Macaranga gigantean* or *Cratoxylum* spp. expressed their highest growth rates between 15 and 22 cm (Karyati *et al.* 2017). Consistent with the lower than expected diameter of maximum growth rate in *A. africana*, we also found that the maximum growth was far lower than many other tropical species (Karyati *et al.* 2017). Altogether, this slower growth of our study species may be explained by the harsh growing environment in which this species occurs where it is restricted to poor and rocky soils (Sinsin *et al.* 2004; Orwa *et al.* 2009).

### Effects of debarking and pruning on growth rates

We tested for the effect of human disturbance on tree absolute growth rate and found that our study species responds differently to several types of disturbance. For example, trunk debarking had no significant effect on tree growth. However, pruning had negative effect on absolute growth rates (Fig. 2), indicating a significant difference in the compensatory tree response to biomass loss depending on plant organs that are targeted. Consistent with this differential response of plant’s organs to disturbance, Ticktin (2004) showed that bark and root removal tend to have more effect on plant demography than leaf harvest. However, in this study, we found an opposite trend. The tolerance of *A. africana* to debarking could be driven by disturbance-induced increase in photosynthetic rates, readjustment and reallocation of resource to compensatory growth (Caldwell *et al.* 1981; Hamerlynck *et al.* 2016). Similar results have been found in *Khaya senegalensis*, where the tree can quickly recover from partial debarking (Gaoue and Ticktin 2007) while the 100 % ringbarked trees will die (Delvaux *et al.* 2009) suggesting that the intensity of bark removed affects plant responses to debarking. *Afzelia africana* is one of the tropical species that exhibited the highest ecological resilience to debarking under stressful arid and semi-arid climate in Benin (Delvaux *et al.* 2010), and this may explain its wide distribution across disturbance and climate gradient in Benin (Orwa *et al.* 2009; Mensah *et al.* 2014). The lack of clear and significant effect of debarking on tree growth may suggest that current bark harvesting intensities might be sustainable (N’Dri 2012; Amahowe *et al.* 2017). This contrasts with results from previous studies which suggest that the growth rates of many other species in West Africa, i.e. *Prunus africanus*, *Grevillea* spp. and *Mondia whytei*, are negatively impacted by debarking (Vuyiya *et al.* 2014). Debarking often breaks some xylem vessel elements that would consequently affect water and nutrient transportation from soil interface to stem and leaves (Maherali and DeLucia 2000; Schreiber *et al.* 2015). This would subsequently exert a negative effect on stomatal conductance and carbon gain for photosynthesis (Panek 1996; Aasamaa *et al.* 2001) and resources to be allocated for growth. Further investigations are needed to understand why this is apparently not the case for *A. africana*.

Contrary to bark removal, pruning reduced diameter growth rate (Fig. 3). Similarly, several studies reported chronic defoliation-driven reduction in diameter growth (Sydnor and McCartney 1996), which would affect carbon sequestration and storage. Pruning *A. africana* is an important activity for cattle breeders because of their high leaf nitrogen content (Orwa *et al.* 2009). As a result, outside of protected areas most individual trees are severely pruned (Ouédraogo-Koné *et al.* 2006; Amahowe *et al.* 2017). Defoliation obviously reduces photosynthesis, hence affecting carbon storage, which can lead to reduced stem growth. Moreover, leaf removal and associated nitrogen loss can affect tree stoichiometry (Beadle 1966; Gaoue *et al.* 2011), and the high cost of post-harvest leaf replacement can shift biomass allocation patterns (Chapin 1980; Dianda *et al.* 2009; Zhou *et al.* 2014). This can ultimately reduce fruit production (Gaoue and Ticktin 2008; Nacoulma *et al.* 2017) and tree population dynamics (Gaoue *et al.* 2013). Environmental conditions including nutrient availability can influence such species response to defoliation (Lovelock *et al.* 1999).

### A weak role for intraspecific trait variability in tree performance

Although several studies have investigated the link between functional traits and plant demography (see Poorter *et al.* 2008, 2010), most studies failed to account for the intraspecific variability in these traits (Burton *et al.* 2017). We explored how intraspecific variation in functional traits predicts growth rate in *A. africana*. Particularly, LMA reflects a trade-off between cheap construction cost and leaf longevity with the fastest growing individuals having the lowest LMA values (Kunstler *et al.* 2016). However, consistent with other studies in tropical regions (Poorter *et al.* 2008; Wright *et al.* 2010), we did not find any significant effect of LMA on tree growth (Table 1, Fig. 4). This lack of relationship between LMA and the absolute growth rate could be due to ontogenetic shifts in biomass investment. However, in a prior study, we found no change in LMA across ontogeny (Amahowe *et al.* 2016) indicating that the biomass investment in leaves per unit of area for *A. africana* is similar across DBH classes. Contrary to LMA, we did find a slight but not significant effect of WD on growth rates (Table 1). Wood density is an indicator of a trade-off in stems between growth performance and strength in stem (Kunstler *et al.* 2016). Indeed, high WD trees are often associated with a slow potential growth rate, while low WD trees are usually associated with rapid growth rate (Herault *et al.* 2010). Individuals with light wood grow faster simply because they produce more volume per unit of biomass (King *et al.* 2006; Poorter *et al.* 2008). There is a body of evidence that individuals with low WD have larger vessels diameter and hydraulic conductance favouring the sapwood efficiency (Baraloto *et al.* 2010; Poorter *et al.* 2010; Hoeber *et al.* 2014) and thus increasing the carbon gain that would be allocated to growth (Santiago *et al.* 2004). However, the global weak relationship between intraspecific functional traits and growth rate in *A. africana* trees may be due to the additional effect of disturbance.

### Individual functional strategy mediates the tree response to disturbance

We demonstrated that WD strongly mediates the tree growth response to pruning (Table 1, Fig. 6). Low WD trees were strongly more affected by pruning, especially at intermediate ages and individuals with high WD tolerated pruning more than those with low WD. Furthermore, we predict that trees with WD > 0.73 g cm^−3^ will incur no pruning-related reduction in growth rate (Fig. 6). This result shows strong evidence of a functional trade-off between WD and response to pruning. Low WD trees tend to grow faster but experience high growth costs when pruned while trees with high WD grow at a slower rate but experience low costs after pruning. High WD trees should thus be selected in high-pruning-pressure environment and vice versa. We further tested this hypothesis splitting our data between trees sampled in forest reserves with low pruning pressure and trees sampled outside forest reserves where pruning intensity is high. Wood density was lower in trees found within forest reserves, and this provides a compelling example of an individual-level adaptation to disturbance.

Internal physiological mechanisms are activated in trees in order to survive and recover from severe disturbance (Vargas 2012). Individual trees with high WD have important resource reserves, i.e. carbohydrates, that could be used not only to compensate for biomass loss (Würth *et al.* 2005), but also to continuously allocate resources to growth in stressful conditions (McDowell *et al.* 2011). Numerous studies provide support for the postulate that stored resources including carbohydrate and nitrogen allow defoliated trees to maintain their growth (Gleason and Ares 2004; Myers and Kitajima 2007; Genet *et al.* 2009). The importance of carbohydrate storage may depend on the tree life history and the local environment. Carbohydrate contents are often lower in dry forest species than in moist ones, where carbohydrate concentration declines with light requirement (Poorter and Kitajima 2007). Given that *A. africana* is a shade-tolerant species in the early stages (Orwa *et al.* 2009; Biaou *et al.* 2011), we expect that carbohydrate storage would be particularly important for this species. Our results emphasize the importance for plant functioning on the interplay between photosynthesis activity and stored resource mobilization (Richardson *et al.* 2013).

The negative effect of pruning on the absolute growth rate suggests that *A. africana* requires increasing attention from forest managers to limit over-exploitation. The unique resilience observed with individuals of high WD provides insights for the phenotype of tree to be selected as good candidates for sustainable pruning. For instance, due to the great socio-economic impact of this activity (Sinsin *et al.* 2004; Ouédraogo-Koné *et al.* 2006), it is unrealistic to prevent Fulani harvesters from pruning the tree; however, forest management programmes can encourage harvesters to target individual trees with high WD which are known to be the larger individuals. Preferably harvesting larger trees can limit the overall negative demographic cost of pruning because our results showed that these individuals are more resilient to harvest.

In addition, for another fodder tree species in our study region, *K. senegalensis*, the Fulani adopt a traditional sustainable management practice, which consists of leaving the top branches (known locally as ‘*Sopoodu*’) during pruning (Gaoue and Ticktin 2009). This *Sopoodu* practice can also be encouraged for *A. africana* trees to ensure the viability of this species.

## Conclusions

This study investigates the effect of intraspecific functional trait variation on the growth rate of a tropical tree species and how it responds to anthropogenic disturbance. The compensatory response of *A. africana* to debarking is good news for its sustainable management. However, ring barking should be avoided to allow the recovery of the damage. Environmental education sessions should be implemented to enhance traditional healers and local communities’ awareness on the sustainable debarking method on woody species. The negative impact of pruning on the absolute growth rate of *A. africana* is highlighted in this work, thus emphasizing the importance of leaving significant part of foliage and branches on tree to allow photosynthesis and subsequently improve tree growth. Furthermore, our research illustrates a strong evidence of a functional trade-off between WD and response to pruning. The cost of being pruned for low dense trees is higher than the cost for trees of high WD, suggesting a resilience strategy of *A. africana* by mobilizing stored resources in stem wood to be reinvested for growth under severe disturbances.

## Sources of Funding

This work was financially supported by a grant from the International Foundation for Science (Grant N° D/5623-1) and IDEA Wild to I.O.A. O.G.G. was supported by a start-up grant from the University of Tennessee, Knoxville.

## Contributions by the Authors

I.O.A., O.G.G. and B.H. conceived the idea and designed the research project. I.O.A. and A.K.N. collected the data. B.H., I.O.A., C.P. and I.C.Z. performed the statistical modelling. I.O.A. drafted the initial manuscript with contribution from O.G.G. and B.H. All the authors contributed critically to the discussion and edited the manuscript before submission.

## Conflict of Interest

None declared.
